# Repair of long bone defects with demineralized bone matrix and autogenous bone composite

**DOI:** 10.4103/0019-5413.80040

**Published:** 2011

**Authors:** Mehmet T Ozdemir, Mustafa Ç Kir

**Affiliations:** Department of Orthopedics, Corlu Military Hospital, Corlu, Tekirdag, and; 1 Department of Orthopedics and Traumatology, Mehmet Akif Ersoy Medical Academy of Cardiovascular Surgery, Istanbul

**Keywords:** Bone healing, bone defect, bone graft, fracture union

## Abstract

**Background:**

Repair of diaphyseal bone defects is a challenging problem for orthopedic surgeons. In large bone defects the quantity of harvested autogenous bone may not be sufficient to fill the gap and then the use of synthetic or allogenic grafts along with autogenous bone becomes mandatory to achieve compact filling. Finding the optimal graft mixture for treatment of large diaphyseal defects is an important goal in contemporary orthopedics and this was the main focus of this study. The aim of this study is to investigate the efficacy of demineralized bone matrix (DBM) and autogenous cancellous bone (ACB) graft composite in a rabbit bilateral ulna segmental defect model.

**Materials and Methods:**

Twenty-seven adult female rabbits were divided into five groups. A two-centimeter piece of long bone on the midshaft of the ulna was osteotomized and removed from the rabbits’ forearms. In group 1 (*n*=7) the defects were treated with ACB, in group 2 (*n*=7) with DBM, and in group 3 (*n*=7) with ACB and DBM in the ratio of 1:1. Groups 4 and 5, with three rabbits in each group, were the negative and positive controls, respectively. Twelve weeks after implantation the rabbits were sacrificed and union was evaluated with radiograph (Faxitron), dual-energy x-ray absorptiometry (DEXA), and histological methods (decalcified sectioning).

**Results:**

Union rates and the volume of new bone in the different groups were as follows: group 1 - 92.8% union and 78.6% new bone; group 2 - 72.2% union and 63.6% new bone; and group 3 - 100% union and 100% new bone. DEXA results (bone mineral density [BMD]) were as follows: group 1 - 0.164 g/cm^2^, group 2 - 0.138 g/cm^2^, and group 3 - 0.194 g/cm^2^.

**Conclusions:**

DBM serves as a graft extender or enhancer for autogenous graft and decreases the need of autogenous bone graft in the treatment of bone defects. In this study, the DBM and ACB composite facilitated the healing process. The union rate was better with the combination than with the use of any one of these grafts alone.

## INTRODUCTION

Repair of diaphyseal bone defects is a challenging problem for orthopedic surgeons. Cancellous autogenous bone is considered as the gold standard for bone grafting applications, but it has some drawbacks for clinical applications, such as limited availability, morbidity, and donor site pain.^1^,^2^ Many graft materials have been used as substitutes for autogenous bone graft (ABG). One of these is demineralized bone matrix (DBM), which is a proven material for bone grafting procedures. It contains collagen as well as noncollagenous proteins like bone morphogenetic proteins (BMP), transforming growth factor-α (TGF-α), and fibroblast growth factor (FGF).^3^,^6^ The osteoinductive properties of DBM causes differentiation of mesenchymal stem cells to the osteoblastic pathway, chemotaxis of osteoblastic precursor cells to the grafted area, and induction of osteoblast function in the grafted area.^7^–^13^ With the use of DBM it may be possible to decrease the amount of ABG in grafting procedures.

Composite grafts have been recently used for the purpose of achieving optimal bone healing response. Combinations of demineralized bone and ABG are already being used in spinal fusions and in diaphyseal long bones.^14^–^16^ Thus, use of the composite would mean less morbidity, potentiation of the healing response at the grafted area, and shortening of bone healing time. We aimed to evaluate a composite of DBM and ABG in the ratio of 1:1 in rabbit diaphyseal bone defects.

## MATERIALS AND METHODS

The allogenic rabbit demineralized bone graft and autogenous rabbit bone graft were used in 2-cm-long rabbit bilateral ulna defect model.^17^ Twelve weeks after graft implantation the extremities were investigated by quantitative and qualitative means for evaluation of defect healing.

Bones were harvested from New Zealand white rabbits and processed according to Reddi-Huggins.^18^ The diaphysis of long bones was cleansed of marrow. Dried bone was pulverized to obtain particle size of 300–800 μm. Demineralization was done with 0.5 M HCl. Grafts were sterilized with ethylene oxide at 38°C before use (Steri-Vac™ 4XL gas sterilizer, 3M Company, St. Paul, Minnesota, USA).

### Design of study

Permission (No. 2001-441) for the study was obtained from the ethical committee of our institution. The study was conducted on 27 adult White New Zealand male rabbits, which were divided into five groups. Groups 1, 2, and 3 had seven rabbits each, while groups 4 and 5 had three rabbits each. In group 1 the defects were treated with ABG, in group 2 with DBM, and in group 3 with a composite of ABG and DBM. In group 4, defects were created but were not treated; this served as the negative control. In group 5, the ulna was left intact and this group served as the positive control.

**Figure 1:**
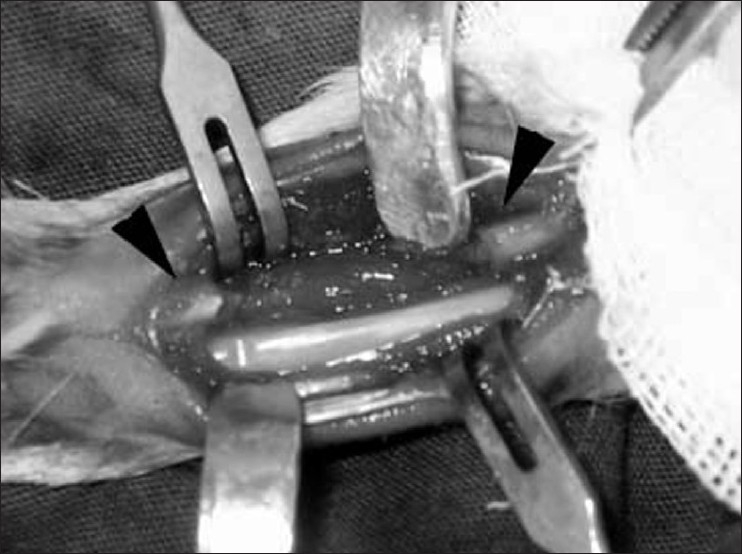
Intraoperative photograph shows a 2-cm-long defect in the ulna with intact radius. Arrowhead indicate the osteotomy sites in the ulna

Bilateral rabbit ulna defect model was used for this study. Crystalline penicillin was first administered at a dose of 5 mg/kg for prophylaxis. Rabbits were anesthetized with ketamine (Ketalar^®^, Pfizer)-xylazine (Rompun^®^, Bayer) and maintained with halothane (Halotan^®^, Turk Hoechst) inhalation. Under aseptic conditions the lateral approach to the forearm was used. A 2 cm-long defect was created on the ulna with osteotomy by connecting drill holes. From the iliac bone 4 cm^3^ of ABG material was harvested and morselized into pieces of about 2 mm using a rongeur. The defects were filled with graft material to obtain complete bridging and filling of the entire defect volume [Figure 1].

Approximately 4 cm^3^ of ABG for group 1, 200 mg of DBM for group 2, and 2 cm^3^ of ABG plus 100 mg of DBM (in the ratio of 1/1) for group 3 were used. The rabbits received a second dose of penicillin G after surgery. Post surgery the animals were provided a warm environment (about 20°C) and housed in individual cages they were well hydrated and were fed mainly fragrant herbs and fresh greens. Sutures were checked daily. Full weight-bearing of forearms, without external or internal fixation, was allowed.

### Evaluation

Twelve weeks after implantation, the rabbits were sacrificed and union was evaluated by qualitative and quantitative means. The methods included: A) gross evaluation, B) high-resolution radiographs, C) dual-energy x-ray absorptiometry (DEXA), D) histological methods (decalcified sectioning).

#### Gross evaluation

The soft tissues were dissected away from the entire forearm and the defected area was examined for cortex formation and the type of tissue that had invaded the defect area.

#### High-resolution radiographs (Faxitron)

For final radiological evaluation, high-resolution radiographs were taken (Mammomat 3, Siemens Electronics, Germany). Radiographs were evaluated according to the Lane scale for two parameters: defect healing (union) and new bone formation in the defect (volume).^19^ Union: New bone formation must bridge with host bone on both sides of the defect. Volume: Percentage of new formed bone to entire defect is scaled as 0% to 100%, then graded with each 25% increments and pointed in five groups as 0 to 4 points.

#### Bone defect union

A defect is considered healed if osseous continuity was restored greater than 25% cross sectional diameter of the defect area. Volume: bone formation within the defect on six point validated scale as follows 0=0%, 1=less than 25%, 2=25% to 49%, 3=50% to 74%, 4=75% to 99%, 5=100%

#### Bone mineral densitometry

Bone density at the grafted area was determined by DEXA (Lunar^®^ACT, Lunar Corporation, USA) and using the software for small animals. First, the whole forearm was scanned and the grafted area was determined (approximately 2 cm long and 0.5 cm wide). DEXA analysis was then carried out to measure the bone mineral density (BMD, in g/cm^2^) in the marked area.^20^

#### Histology

Extremities were fixed in 70% formaldehyde for 7 days and decalcified in 20% formic acid for 6 days. Sagittal sections (5-μm thick) of the forearm were made, and at least three samples were collected for each extremity. Sections were stained with hematoxylin-eosin and Masson trichrome. Evaluation was carried out by two independent observers.^21^

### Statistical analysis

For statistical analysis, NCSS (Number Cruncher Statistical System) for 2007 and PASS 2008 statistical software (Utah, USA) were used. Descriptive statistics (calculation of median and mean with standard deviation), oneway ANOVA test, and Tukey HSD test were used as appropriate. 95% confidence intervals were estimated; P≤.05 was considered as statistically significant.

## RESULTS

One rabbit from group 2 died in the early postoperative period. One from group 2 suffered from a left radius fracture and this extremity was excluded from the experiment. The other rabbits survived without any complication (including infection) at the grafted area or the donor site (the iliac bone) until the end of 12 weeks. A total of 14 extremities in group 1, 11 in group 2, and 14 in group 3 were observed during the study.

### Gross evaluation

**Figure 2:**
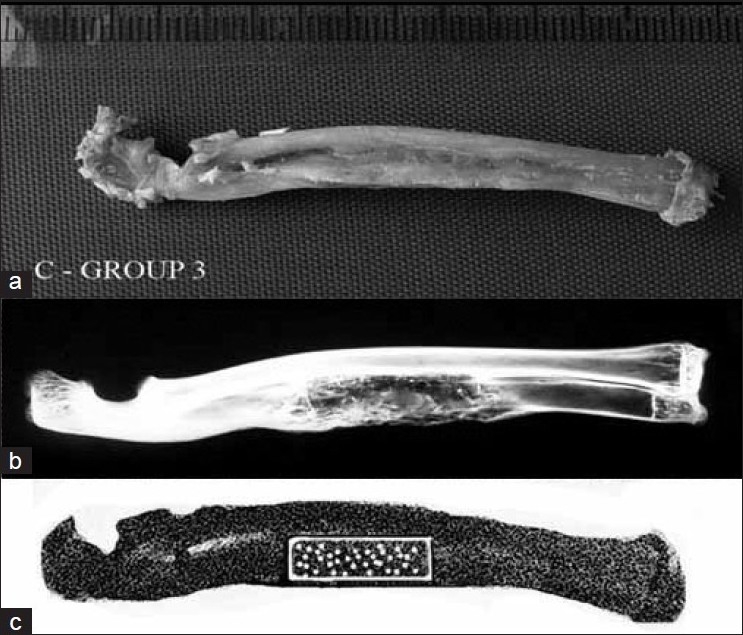
(a) The radiograph shows healed defect in an animal of group 3. (b) Gross specimen shows healed defects of the ulna. (c) Scanned whole forearm and marked area, which represents the healed defect, are seen at densitometric measurement

Observations on gross evaluation showed correlation with the radiographical findings. Sufficient new bone formation and bridging with the host bone on both sides of the defect was visible in groups 1, 2, and 3. Rigid synostosis was observed between the radius and the ulna alongside the grafted area. New bone formation in group 2 was less than that in groups 1 and 3. Corticalization and volume of new bone was obviously superior in group 3. Healed defects were grossly stable in all groups. Fibrous tissue formation was seen in the defected area, which was especially prominent in group 4 [Figure 2a].

### Radiological results

Defect area filled by new bone is evaluated radiographically for groups 1,2,3 after 6 weeks. After this period, incorporation at the grafted area was more prominent in group 3 [Figure 2b] than in groups 1 and 2. High radiodensity and new bone formation was more clearly evident at this stage of the healing process in group 3 than in groups 1 and 2. Defect healing rate is gradually increased throughout the control period. After 12 weeks, new bone formation in groups 1, 2, and 3 was sufficient to unite and bridge the defect. A defect was considered healed if new bone formation occupies more than 25% of the defect volume.^19^ Radiological assessment according to the Lane scale (union and volume of the new bone) was performed by two independent observers.^19^ The results were as follows: group 1 (14 extremities) - 92.8% union and 78.6% new bone, group 2 (11 extremities) 72.2% union and 63.6% new bone, and group 3 (14 extremities) - 100% union and 100% new bone. There was no evidence of new bone formation in group 4 during the experiment period.

### Bone mineral densitometry

The mean score of each group was determined and recorded. Group 3 (DBM and ABG composite) had the highest densitometric score. The average DEXA results for all limbs in each group were as follows: group 1 (14 extremities): BMD = 0.164 g/cm^2^, group 2 (11 extremities): BMD = 0.138 g/cm^2^, and group 3 (14 extremities): BMD = 0.194 g/cm^2^ [Figure 2c]. The increase in group 3 was significantly more than that in group 2 and group 1 (*P*=.001 and *P*=.001, respectively), and the increase in group 1 was statistically significant as compared to that in group 2 (*P*=.001).

### Histology

Histological results correlated well with the radiographical findings. Healing was significantly better in group 3. In group 1 new bone formation was largely lamellar, but some areas were filled with primary bone; bone marrow was evident at the whole area. Corticalization and union at the bonding edges was grossly sufficient. In group 2, primary bone was surrounded by lamellar bone. The volume of new bone formation was sufficient but corticalization and union was scanty in some specimens. Bone marrow elements entirely filled the area in the ABG treated group.

**Figure 3:**
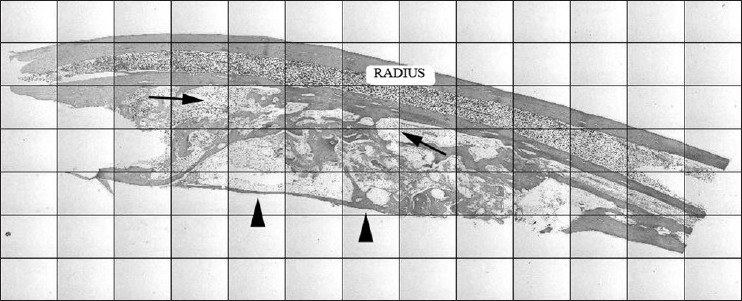
Histologic sections of the grafted area and union sites. Black arrowheads indicate corticalization at the grafted area; black arrows indicate bone marrow between the osteoid of newly formed bone. The intact radius is also seen

In group 3, the new bone volume at the grafted area was sufficient. Corticalization was similar to that in group 1. The vascularization pattern of new bone was homogenous as in group 2. The ratio of primary bone to lamellar bone was weighted in favor of lamellar bone. In group 4 the defected area was filled with fibrovascular tissue. There was scant bone formation close to the radius [Figure 3].

## DISCUSSION

The unique properties of autogenous bone make it the gold standard for grafting procedures. In the last two decades, demineralized bone has begun to be used as an alternative to autogenous bone because it provides spectacular bone healing response. Repair of diaphyseal defects has been attempted with both ABG and DBM. The reported union rates with ABG are variable, being between 40%-100%. Low union rates are caused with use of cortical grafts or syngeneic grafts, but when cancellous bone is used the union rates are always around 90%.^3^,^7^,^13^,^19^,^22^ The union rates with DBM are also variable, being between 60%–100%.^1^,^3^,^7^,^8^,^13^,^22^–^24^ Although the differences in union rates are believed to be related to the type of manufacturing process of DBM, this has not been definitely established because of difficulties in quantitative measurement of the efficiency of demineralized bone.^25^ Our study found 92% and 72% union rates for ABG and DBM respectively.

In the past, efforts have been made to facilitate the bone healing response of DBM via composition with various graft materials.^1^,^2^,^8^,^13^,^24^ These attempts were due to reports regarding the insufficiencies of DBM. Due to rapid release of osteoinductive factors by DBM, the bone healing response may not be constant during the entire healing process.^9^,^24^,^26^ For maintaining the concentration of these factors some matrices have been added to the structure of DBM.^22^ Solheim *et al*. used DBM alone as well as in combination with biodegradable polyorthoester in radius segmental defect model in rats. They found that the combination of polyorthoester + DBM resulted in production of significantly higher volume of new bone than with DBM alone.^24^ DBM has little osteoconductivity and its lack of mechanical integrity discourages its use in areas where structural support is essential. Therefore osteoconductive matrices like tricalcium phosphate and hydroxyapatite are composited with DBM.^21^ Lack of osteogenic cells in DBM has led to combination of DBM with living cells and addition of bone marrow into DBM. Wittbjer and Gebhart used DBM and autologous bone marrow in diaphyseal long bone defects. Their findings proved that bone formation was induced and mineralization was accelerated at the grafted area with this composite.^10^,^22^

ABG and DBM composite are used for vertebral fusions and craniofacial defects.^5^,^11^,^27^ With the composite union rates and the volume of the healed defect is 178% better than with DBM alone.^20^ Maturation of new bone was observed earlier in the composite group. Consequently, DBM has gained a reputation as a ‘graft enhancer and extender’.^10^ It is clear that DBM potentiates the effect of ABG.

Synergism between DBM and ABG may be due to rapid revascularization providing an excellent graft bed, enhancement of endochondral ossification, and osteoinduction by specific peptides.^27^ DBM causes rapid revascularization through the graft bed, thus enhancing the bone healing response. DBM provides a better osteoinductive environment than fracture hematoma for ABG. It fills the spaces between the particles of autogenous bone chips and thus the osteoinductive factors in DBM easily reaches the osteoblastic precursors.^11^ Furthermore, the autogenous graft serves as a controlled-release mechanism for the bioactive molecules in DBM, allowing the composite to mimic the natural release rates. DBM induces endochondral new bone formation and this provides an excellent template for the upcoming bone formation by ABG.^5^,^11^,^23^

In our study, when autogenous bone was used alone there was up to 92% union rate, and 72% of the defect volume was filled with new bone. Combination of DBM with ABG provided a rapid and effective bone healing response, with complete reconstruction of segmental bone defects. The radiographical results indicated that the combination of DBM and ABG for grafting increased the volume of new bone formation and bone mineral density at the defected area as compared to grafting with either one alone. Our data suggests that DBM serves as a graft enhancer in diaphyseal defects just as it does in spinal fusions. We did not carry out mechanical failure measurements of healed bones (e.g., bending or rotational stress tests), but, overall, our data suggest that DBM facilitates the effect of ABG and decreases the amount of autogenous bone needed in repair of segmental bone defect and thus also decreases morbidity.
